# An Evaluation of Right-Sided Symptom Onset as a Predictor of Poor Parkinson’s Disease Prognosis

**DOI:** 10.7759/cureus.13493

**Published:** 2021-02-22

**Authors:** Asuman Orhan Varoğlu, Adem Aydin

**Affiliations:** 1 Neurology, Istanbul Medeniyet University, Istanbul, TUR; 2 Neurology, Kastamonu State Hospital, Kastamonu, TUR

**Keywords:** parkinson’s disease, prognosis, clinical symptoms, asymmetry

## Abstract

Objective

The current study aimed to measure and compare neurological disability in Parkinson’s disease (PD) patients with right-sided symptom onset with that in PD patients with left-sided symptom onset, using the measurements taken at their first and last visits, to determine if right-sided symptom onset was predictive of a poor PD prognosis.

Methods

One hundred and forty-three PD patients were included in the study. The Unified Parkinson’s Disease Rating Scale (UPDRS) and the Hoehn and Yahr Scale were used to measure neurological disability in patients at the first and last visits. The scores for the neurological disability of patients at the first and last visits were compared retrospectively relative to disease onset.

Results

Seventy-six PD patients had right-sided symptom onset (53%), and 67 patients had left-sided symptom onset (47%) (*p* = < 0.001). The differences between the scores at the first and last visits, measured using the UPDRS and the Hoehn and Yahr Scale, were higher for PD patients with right-sided symptom onset than patients with left-sided symptom onset (*p *= < 0.001, *p* = < 0.002, respectively). Similarly, the UPDRS Part II and Part III values, used to evaluate motor function, were higher in PD patients with right-sided symptom onset as compared to those with left-sided symptom onset at the first and last visits (*p *= < 0.001).

Conclusion

Right-sided symptom onset was predictive of a poor prognosis in PD patients at follow-up.

## Introduction

Parkinson’s disease (PD) is a chronic neurodegenerative disease that occurs due to the progressive loss of dopaminergic neurons in the substantia nigra. The clinical manifestations of PD often begin asymmetrically. In the absence of motor asymmetry in the clinical symptoms of such patients, the differential diagnosis of the condition should include other diseases [1]. After the loss of neurons (50%-70%) in the substantia nigra, clinical symptoms and signs that are contralateral to the affected substantia nigra are observed [2].

Reasons for asymmetric motor symptoms in PD have been speculated in the literature [3-4]. Toxic or metabolic intoxications, environmental exposure, or genetic or structural changes are thought to cause predominantly unilateral motor manifestations. The number of dopaminergic neuron cells in the right and left hemispheres of healthy individuals has been reported to differ by 30%. Conversely, such a difference was not reported elsewhere [3-6]. Regardless, the difference in the distribution of dopaminergic neurons between the hemispheres may be insufficient in explaining motor predominance. Consequently, most research has only randomly described motor predominance in patients with PD [1].

Consideration has been given to the prognosis of PD patients, most of which have primarily related to biomarkers or clinical data. Asymmetric brain immune modulation between the hemispheres has been identified in many studies [7-9]. In addition, peripheral asymmetric immune function in various diseases has been demonstrated [10-12]. The first clinical study to demonstrate central immune asymmetry in multiple sclerosis patients was conducted by the authors of the current research [13].

Thus, the current study aimed to measure and compare neurological disability in PD patients with right-sided symptom onset, measured at their first and last visits, with PD patients with left-sided symptom onset, to determine if right-sided clinical signs at onset were predictive of a poor PD prognosis.

## Materials and methods

This study was retrospectively conducted at Istanbul Medeniyet University, Goztepe Research and Educational Hospital, Istanbul, Turkey, and the Department of Neurology, Kastamonu State Hospital, Kastamonu, Turkey. One hundred and forty-three patients with idiopathic PD, aged 40-90 years, were included. Patients with Parkinson-plus syndromes, secondary parkinsonism, destructive lesions caused by other brain disorders, and other neurological diseases were excluded. The outpatient records of patients who presented between 2015 and 2020 were included.

The United Parkinson’s Disease Rating Scale (UPDRS) was used to evaluate the clinical status of the patients [14]. The scores obtained using the UPDRS and Hoehn and Yahr Scale for PD patients with right-sided symptom onset, measured at their first and last visits, were compared with those of patients with left-sided symptom onset. Thus, information about disease prognosis was evaluated according to the side on which the clinical signs first manifested. The first and last presentations of all patients were evaluated by the authors themselves. The UPDRS and Hoehn and Yahr Scale scores were assessed in relation to the age, gender, disease duration, and hand dominance of the patients.

Institutional ethics committee approval was obtained from the Karabuk University Training and Research Hospital Clinical Research Ethics Committee (date/protocol: 2020/322).

Statistical analysis

Statistical analysis was performed using the Statistical Package for the Social Sciences (SPSS® version 22) (IBM Corp., Armonk, NY). Normal distribution of the variables was evaluated using visual (histogram and probability graphs) and analytical methods (Kolmogorov-Smirnov and Shapiro-Wilk tests). Descriptive analysis was performed, and the median and interquartile range (and frequency tables for the ordinal variables) was used to describe the non-normally distributed variables. The relationship between the mean and median values of the UPDRS and Hoehn and Yahr Scale scores obtained at the first and last visits of the patients and the side on which the clinical symptoms manifested at onset was evaluated using the Mann-Whitney U and Wilcoxon signed-rank tests. In addition, the relationship between the UPDRS and Hoehn and Yahr Scale scores obtained at the first and last visits was evaluated in relation to the age, gender, disease duration, and hand dominance of the patients using Spearman’s rank-order correlation coefficient. A p-value of <0.05 was considered statistically significant.

## Results

There were 59 (41%) female and 84 (59%) male patients. The patients were aged 34-93 years (mean of 67.48 ± 10.9 years). The disease duration ranged from two to 20 years (mean of 6.65 ± 7.34 years). The average follow-up was 3.41 + 0.98 years. Seventy-six patients had right-onset PD (53%) and 67 patients had left-onset PD (47%) (p < 0.001), and this finding was statistically significant (p < 0.001). The mean age of PD onset was 61.06 ± 10.75 years. At both the first and last visits, the UPDRS and Hoehn and Scale scores were higher in patients with right-sided symptom onset than those in patients with left-sided symptom onset (p < 0.001). In addition, the differences between the scores at the first and last visits, measured using the UPDRS and the Hoehn and Yahr scale, were greater in PD patients with right-sided clinical signs at onset as compared to those with left-sided clinical symptoms at onset (p < 0.002). Similarly, the UPDRS Part II and III values, used to evaluate motor function and measured at the first and last visits, were higher in PD patients with right-sided symptom onset as compared to those with left-sided symptom onset (p < 0.001) (Table 1). A correlation was not found between the side of onset and disease duration using Spearman’s rank-order correlation coefficient.

**Table 1:** Baseline characteristics and change variables according to Parkinson disease onset side © One-way ANOVA test used, #Mann-Whitney U test used, *Mean Values, ¥ Median Values, D Duration: Disease Duration, F: First, L: Last, D: Difference, §: Score, π: Fisher exact test used, ª: T-test used, €: Wilcoxon signed-rank test used. F- UPDRSpart II: First UPDRS - part II, F - UPDRSpart III: First UPDRS - part III, L - UPDRSpart II: Last UPDRS part II L -UPDRSpart III: Last UPDRS part III, D - UPDRS: (difference last - first UPDRS). D-HoenhYahr: (difference last - first HoenhYahr) ANOVA: analysis of variance

ONSET SIDE										
	Case (n)		R			L			p	
Case (n)	143 (100%)		76 (53.1%)			67 (46.9%)			0.001^ª^	
Age*¥	67.48±10.9*	69 (34-93)^¥^	67.64 ± 11.27*	70(34-87)^¥^	67.30±10.68*		69(44-93)^ ¥^	0.851^©^		
Starting Age	61.06±10.75*	62 (31-89)^ ¥^	60.97 ± 10.75*	61 (31-80)^ ¥^	61.16 ± 10.84*		63(38-89)^¥^	0.916^©^		
D Duration	6.09 ± 3.04*	6(2-20)^ ¥^	6.53 ± 3.35*	6 (1-20)^ ¥^	5.5 ± 2.41*		5 (2-14)^ ¥^	0.077^©^		
Gender									0.645^#^	
Female	59 (41.3 %)		30 (39.5 %)			29 (43.3%)			0.734^ π^	
Male	84 (58.7 %)		46 (60.5 %)			38 (56.7%)				
T	143(100 %)		76 (53 %)			67 (47 %)				
F-UPDRS^§^	40^¥^		44^¥^			25^¥^			0.001^€^	
L-UPDRS^§^	51^¥^		56^¥^			30^¥^			0.001^€^	
D-FUPDRS^§^	9^¥^		11^¥^			5^¥^			0.001^€^	
F-HoenhYahr^§^	2^¥^		2.5^¥^			2^¥^			0.001^€^	
L-HoenhYahr^§^	3^¥^		3 ^¥^			2^¥^			0.001^€^	
D-HoenhYahr^§^	0. 5		0.5			0			0.002^€^	
F- UPDRSpart II	7^¥^		7			4			0.001^€^	
F-UPDRSpart III	22^¥^		26			14			0.001^€^	
L-UPDRSpart II	9^¥^		10			4			0.001^€^	
L-UPDRSpart III	26 ^¥^		28			17			0.001^€^	

When hand dominance was evaluated, the age of PD onset was lower in left- than in right-handed patients (p < 0.001), and PD was more common in right- than in left-handed patients (p < 0.001). Among the left-handed patients, PD was slightly more common in women (n = 21, 36%) than men (n = 9, 11%). At both the first (p < 0.012) and last visits (p < 0.004), the UPDRS scores were higher in left- than in right-handed patients. There were no differences in D-UPDRS (difference last visits - first visits UPDRS) scores between right-handed and left-handed patients (p > 0.05). Conversely, the difference between the first visits and last visits scores was higher in right- than in left-handed patients using the Hoehn and Yahr Scale (p < 0.007) (Table 2). A positive correlation was observed between the side of disease onset and hand dominance using Spearman’s rank-order correlation coefficient. PD patients with right-sided symptom onset tended to be right-handed, and PD patients with left-sided symptom onset tended to be left-handed, using the chi-square test (p = 0.001).

**Table 2:** Baseline characteristics and change variables according to hand dominance © One-way ANOVA test used, #Mann-Whitney U test used, *Mean Values, ¥Median Values, F: First, L: Last, D: Difference, §: D-UPDRS: (difference last - first UPDRS) Score, ª: T-test used, π: Fisher exact-test used, €: Wilcoxon signed-ranks test used, D Duration: Disease Duration, D-HoenhYahr: (difference last - first HoenhYahr) ANOVA: analysis of variance

HAND DOMINANCE										
	Case (n)		R			L			p	
Case (n)	143 (100%)		113 (79 %)			67 (21 %)			0.001^ª^	
Age*¥	67.48±10.9*	69 (34-93)^¥^	67.64 ± 11.27*	70(34-87)^¥^	67.30±10.68*		69(44-93)^ ¥^	0.851^©^		
Starting Age	61.06±10.75*	62 (31-89)^ ¥^	62.72 ± 9.81*	62 (38-89)^ ¥^	54.83 ± 12.02*		54(31-80)^¥^	0.001^©^		
D Duration	6.09 ± 3.04*	6(2-20)^ ¥^	6.26 ± 3*	6(2-20) ^¥^	5.4 ± 3.17*		5(2-6)^ ¥^	0.737^©^		
Gender									0.001^#^	
Female	59 (41.3 %)		38 (64.4 %)			21 (35.6%)			0.001^π^	
Male	84 (58.7%)		75 (89.3 %)			9 (10.7%)				
T	143 (100%)		113(79 %)			30 (21 %)				
F-UPDRS^§^	40^¥^		34^¥^			45^¥^			0.012^€^	
L-UPDRS^§^	51^¥^		47^¥^			59^¥^			0.004^€^	
D-UPDRS^§^	9^¥^		8^¥^			9^¥^			0.212^€^	
F-HoenhYahr^§^	2^¥^		2^¥^			2.5 ^¥^			0.476^€^	
L-HoenhYahr^§^	3^¥^		3^¥^			3^¥^			0.063^€^	
D-HoenhYahr^§^	0.5		1			0.5			0.007^€^	

PD was more common in men (p < 0.001). At both the first (p < 0.013) and last visits (p < 0.006), the UPDRS scores were higher in female patients. A difference was not observed at any time in terms of the Hoehn and Yahr scale scores according to gender (Table 3). In the current study, most right-handed patients were male, and most left-handed patients were female, using the chi-square test (p = 0.001).

**Table 3:** Baseline characteristics and change variables according to gender © One-way ANOVA test used, #Mann-Whitney U test used, *Mean Values, ¥Median Values, F: First, L: Last, D: Difference, §: Score, ª: T-test used, π: Fisher exact test used, €: Wilcoxon signed-rank test used, D Duration: Disease Duration ANOVA: analysis of variance

HAND DOMINANCE								
	Case (n)		R			L		p
Case (n)	143 (100%)		59 (41.3 %)			84 (58.7%)		0.001^#^
Age*¥	67.48±10.9*	69 (34-93)^¥^	67.74 ± 10.39*	69 (48-92)^¥^	67.29 ± 11.39*		69.5 (34-93)^ ¥^	0.811^©^
Starting Age	61.06±10.75*	62 (31-89)^ ¥^	61.16 ± 10.57*	60 (38-89)^ ¥^	60.98 ± 10.75*		62.5 (31-80)^¥^	0.921^©^
D Duration	6.09 ± 3.04*	6(2-20)^ ¥^	5.81 ± 2.45*	5 (2-12)^ ¥^	6.23± 3.43*		6 (2-20)	0.457^©^
F-UPDRS^§^	40^¥^		42 ^¥^			33 ^¥^		0.013^€^
L-UPDRS^§^	51 ^¥^		58 ^¥^			45 ^¥^		0.006^€^
D-FUPDRS^§^	9 ^¥^		9^¥^			8^¥^		0.236^€^
F-HoenhYahr^§^	2^¥^		2.5^¥^			2^¥^		0.159^€^
L-HoenhYahr^§^	3^¥^		3^¥^			3^¥^		0.075^€^
D-HoenhYahr^§^	0.5		0.5			0.5		0.063^€^

## Discussion

Differences in brain immune function between the hemispheres have been demonstrated in animal studies [7-15]. Functional and anatomical differences between the hemispheres have also been demonstrated [16-17]. Many clinical parameters have been evaluated in prognosis studies on patients with PD [18-20]. However, to the best of our knowledge, the impact of the asymmetrical presentation of clinical symptoms on the prognosis of patients with PD has not previously been evaluated, although many researchers believe that motor predominance is essential for a diagnosis of parkinsonism.

The current study investigated the effect of asymmetric symptom onset on the prognosis of PD patients. Specifically, right-sided symptom onset negatively impacted the disease prognosis, both at the onset of the disease and later. The differences between the scores, measured during the UPDRS and the Hoehn and Yahr scale at the first and last visits, were greater in PD patients with right-sided symptom onset as compared to those with left-sided symptom onset.

Higher UPDRS values were observed at both the first and last visits of patients with right-sided clinical signs at onset, especially for motor function (Part II and Part III), as compared to patients with left-sided clinical signs at onset. In addition, disease duration was higher in patients with right-sided symptom onset. Thus, right-sided symptom onset in PD patients was shown to negatively impact the functional capacity of patients, both at onset and later. These findings indicate that the nigrostriatal pathway in the left hemisphere is more sensitive to disturbance than the right hemisphere.

It is well-known that brain modulation of the immune system is asymmetric [9]. Increasing the level of interleukin-1 (IL-1) and interleukin-6 (IL-6) induces the hypothalamic-pituitary-adrenal axis [21-23]. It has been demonstrated that IL-1 is involved in brain immune modulation to a greater extent than IL-6 [15]. IL-1 and IL-6 are produced more abundantly in the right hemisphere of the brain as compared to the left [24]. In one study, T-cell activity and natural killer (NK) cell activity decreased significantly with left hemisphere ablation, but the same effect was not seen with right hemisphere ablation [25]. This shows that much more serious functional losses may be caused by left than right hemisphere lesions due to the decrease in NK and T cells in the latter. This elucidates the greater severity of right-sided clinical manifestations, compared to left-sided clinical manifestations, at both the first and last visits in the current study, indicative of the loss of left hemisphere function.

The effect of hand dominance on motor function and its relationship with the disease has been described elsewhere [12,26]. In the current study, the number of right-handed patients was statistically higher than the number of left-handed patients. In most right-handed patients, the left hemisphere is the dominant hemisphere [27-28]. Therefore, in the current study, it was unsurprising that PD was more common in right-handed patients since the left hemisphere is more vulnerable to disturbance than the right hemisphere. However, Parkinson’s disease starts at a much earlier age in left-handed patients, and the neurological disability is also severe. The dominant hemisphere is primarily the right hemisphere in left-handed people; however, the left hemisphere can be dominant in 15%-20% of cases [27-28]. The dominant hemisphere and immunological differences in left-handed people may be more complicated than what has been established currently. In the current study, most left-handed patients were also found to be women. The difference between the first and last visits scores was higher in right- than in left-handed patients using the Hoehn and Yahr scale.

A relationship was not found between hand dominance, disease duration, and the age of PD patients. In many diseases, the dominant hemisphere and hand use differ vastly from the healthy population. For example, Lewin et al. reported that Down’s syndrome, epilepsy, and autism patients were characterized by a significant increase in left-handedness and hand use complexity [29]. Pain was frequently experienced on the left side of the head in a study that evaluated migraines in left-handed patients [30]. In the current study, PD was more common in male and right-handed patients, although women and left-handed patients had greater neurological disability assessed using UPDRS scores. Although PD starts worse in left-handed people in terms of neurological disability than right-handed people, the progression rate of the disease in right-handed people (D-HoenhYahr) is higher than in left-handed people. Thus, gender and hand dominance could be factors that affect central asymmetric immune function in PD patients, which is not the case in the healthy population. Although the current study was retrospective and the number of patients included was relatively limited, it is notable that it is the first study to have evaluated the effect of lateralization on the prognosis of patients with PD.

## Conclusions

The right-sided onset clinical findings were negative prognostic factors in patients with Parkinson’s disease. We believe that the presence of right-sided clinical findings at the onset is an important guide in the regulation of follow-up and prognosis and the treatment of these patients.
